# Comparative efficacy of targeted structural patterns of electroencephalography neurofeedback in children with inattentive or combined attention deficit hyperactivity disorder

**DOI:** 10.1002/brb3.2572

**Published:** 2022-04-24

**Authors:** Feng‐Hua Wang, Li‐Yan Sun, Xiao‐Mei Cui, He‐Dan Zhao, Ling‐Fei Yang, Zheng Wang, Tong‐Kun Shi

**Affiliations:** ^1^ School of Medicine Jiaxing University Jiaxing China; ^2^ Women's and Children's Hospital Affiliated to Jiaxing University Jiaxing China; ^3^ Jiaxing Kangci Hospital Jiaxing China

**Keywords:** attention deficit hyperactivity disorder, different structural patterns, efficacy, electroencephalography, neurofeedback

## Abstract

**Objective:**

To evaluate and compare the effects of three courses of different structural patterns of electroencephalography neurofeedback on predominantly inattentive attention deficit hyperactivity disorder (ADHD‐PI) and combined ADHD (ADHD‐CT).

**Methods:**

Thirty‐eight ADHD‐PI and ADHD‐CT children were selected and completed three courses of different structural patterns of electroencephalography neurofeedback according to their ADHD type. Before and after each course, relative power value of electroencephalography, including *θ*, *β*, *α*, SMR and their ratios (*θ*/*β*, *θ*/*α*), and eighteen integrated visual and auditory continuous performance test (IVA/CPT) quotients were obtained and compared. Data were analyzed by SPSS software, and *p *< .05 was considered statistically significant.

**Results:**

After one course, *θ*, three IVA/CPT quotients in both types and two comprehensive quotients in ADHD‐CT changed significantly (all *p* < .05). After two courses, *θ*/*α*, *θ*/*β* and five IVA/CPT quotients in both types, *θ* and *α* in ADHD‐PI, four comprehensive quotients, and four respond control quotients in ADHD‐CT varied significantly compared to before treatment and after one course (all *p* < .05). After three courses, *α*, *β*, *θ*, *θ*/*α*, *θ*/*β* and ten IVA/CPT quotients in both types changed significantly compared to before treatment and after one course (all *p* < .05). In addition, six IVA/CPT quotients in both types after three courses were significantly higher than those after two courses (all *p* < .05).

**Conclusion:**

Different structural patterns of electroencephalography neurofeedback targeted for ADHD‐CT and ADHD‐PI were both effective and feasible. Three courses of EEG neurofeedback were most effective.

## Graphical abstract

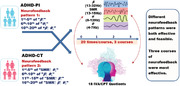

## 1 INTRODUCTION

Attention deficit hyperactivity disorder (ADHD) is one of the most common neurological and behavioral disorders during childhood (Wolraich et al., 2019). A recent study showed pooled worldwide ADHD prevalence of 7.2% among children (Thomas et al., 2015), with estimates from some community‐based samples being somewhat higher at 8.7% to 15.5% (Rowland et al., 2015; Wolraich et al., 2014). Children with ADHD often show signs of distraction, excessive activity, poor impulse control, and poor overall control, which do not correspond to their age of learning or socialization (Wolraich et al., 2019). The personal learning and interpersonal contact abilities of ADHD patients are sometimes seriously affected, and patients can even develop cognitive, conduct, and emotional disorders (Baijot et al., 2017). In the clinic, ADHD is divided into the predominantly inattentive type (ADHD‐PI, 45% of ADHD patients), the predominantly hyperactive/impulsive type (ADHD‐HI, 21% of ADHD patients), and the combined type (ADHD‐CT, 34% of ADHD patients) (Cueli et al., 2019; Valmiki et al., 2021).

Over the past few decades, different treatments and interventions aimed at inattention, hyperactivity, and impulsivity have been used. Medications, including methylphenidate, amphetamine, atomoxetine, and guanfacine, have been widely used in the acute treatment of ADHD (Van Doren et al., 2019) and reported to be effective for some features in short‐term (Lin et al., 2021); however, medications failed to improve mood, self‐esteem, and social relationships (Harpin et al., 2016). Furthermore, the compliance of ADHD patients generally decreases after one year's medications (Frank et al., 2015). In addition, some psychosocial treatments for ADHD children and adolescents, including behavioral therapy (parent training, classroom management, peer interventions), and cognitive training have been demonstrated to be effective (Chan et al., 2016; Che et al., 2021; Evans et al., 2018; Sonuga‐Barke et al., 2013).

Besides medications and psychosocial treatments, neurofeedback is also considered as a valid option (Arns et al., 2020; Enriquez‐Geppert et al., 2019). This treatment method can avoid side effects of drugs and has already gained popularity in recent years (Arns et al., 2014). As a non‐invasive and relatively new approach for treating brain‐related conditions, electroencephalography (EEG) neurofeedback can normalize disrupted brain waves that associate with ADHD by means of repeated training based on largely operant conditioning (Razoki, 2018). Thus, EEG neurofeedback is a method that assists subjects to control and adjust their brain waves (*θ*/*β*/*α*/SMR) consciously (Arns et al., 2013; Clarke et al., 2002; Lubar & Shouse, 1976; Ogrim et al., 2012). Many researchers have gained a positive conclusion about the efficacy of EEG neurofeedback in ADHD (Garcia Pimenta et al., 2021; Nooner et al., 2017). However, there are few studies on the dynamic changes in structural patterns and multiple courses of EEG neurofeedback in the treatment of different types of ADHD.

In this trial, on the one hand, the effects of different structural patterns of EEG neurofeedback targeted for different types of ADHD were evaluated and compared. On the other hand, the dynamic changes in efficacy evaluation indexes among treatment courses were observed to evaluate the long‐term efficacy of multi‐course EEG neurofeedback in the treatment of ADHD, thereby providing a new basis for the establishment of a practical treatment model for children with different ADHD types.

## 2 METHODS

### 2.1 Participants and treatment

From June 2017 to August 2019, children diagnosed with ADHD‐PI, ADHD‐HI, or ADHD‐CT according to the fifth edition of the American Psychiatric Association Diagnostic and Statistical Manual for Mental Disorders (DSM‐V) (American Psychiatric Association, 2013) at the Pediatrics Department of Women's and Children's Hospital Affiliated with Jiaxing University were recruited for this study. At the same time, children with mental retardation (Raven Intelligence Test, IQ < 70), brain injury, mental illness, psychiatric drug use (in the 3 months before recruitment), and the habit of drinking stimulant drinks such as coffee and cola were excluded. In addition, parents who had received educational instruction of behavioral therapy or children who had received training in behavioral therapy themselves were also excluded. Finally, 38 ADHD patients (19 ADHD‐PI and 19 ADHD‐CT) met the inclusion and exclusion criteria and completed three courses of EEG neurofeedback. A total of eight ADHD‐HI children were selected in this study, but only four children completed three courses of EEG neurofeedback. Due to the small sample size and insufficient representation, they were not included in the final statistics and will not be mentioned below.

### 2.2 EEG neurofeedback

The BioNeuro biofeedback instrument from Canadian Thought Technology was used for ADHD treatment, and it measured changes in the relative power of EEG. According to our previous study (Shi et al., 2012) and the internationally accepted 10–20 Electrode Placement System, Cz (positive electrode), right lobe A2 (negative electrode), and left lobe A1 (reference electrode) were selected as the most stable EEG detection areas. Participants were required to wash their hair before testing; Cz, A1, and A2 areas needed to be wiped with alcohol; the head sebum needed to be removed with scrubbing, and the conductive cream needed to be applied to the Cz area before the electrodes were placed.

The test room was indoors, quiet, and with soft lighting. The use of psychostimulant drugs and stimulant drinks were banned 48 h before treatment, respectively. Firstly, subjects were accompanied by a pre‐test instructor and remained in the test room for 10 min to familiarize the surroundings and reduce their tension and anxiety. Thereafter, subjects were instructed to close their eyes and relax for 1 min. Researchers measured the EEG baseline for 3 min, and the treatment threshold was set. According to the protocol, the treatment commenced and researchers guided children to strive for and image the target, and children would be rewarded when the set target was achieved. After treatment, the EEG baseline was measured for 3 min again.

In this study, according to the clinical characteristics of the different types of ADHD patients, different structural patterns of EEG neurofeedback were adopted. The structural patterns of EEG neurofeedback for ADHD‐PI and ADHD‐CT patients were 1st–5th of “*β* ↑”, 6th‐10th of “*θ* ↓”, 10th–20th of “*β* ↑ *θ* ↓” and 1st–5th of “SMR ↑ *θ* ↓”, 6th–10th of “*β* ↑ *θ* ↓”, 11th–15th of “SMR ↑ *β* ↑”, 16th–20th of “SMR ↑ *β* ↑ *θ* ↓,” respectively. Each EEG neurofeedback lasted for 20 min, and a treatment course consisted of 20 repeated EEG neurofeedbacks, three to four times per week. Participants were required to complete three courses continuously. The relative power value of *θ* (4−7 Hz), *β* (13−32 Hz), *α* (8−12 Hz), and SMR (13−15 Hz) were recorded, and the ratios of *θ*/*β* and *θ*/*α* were calculated before and after each treatment course in order to evaluate the efficacy of EEG neurofeedback.

### 2.3 Integrated visual and auditory continuous performance test (IVA/CPT)

According to the literature (Corbett & Constantine, 2006; Kim et al., 2015), IVA/CPT quotients (Braintrain, USA) were adopted to reflect changes in cognitive ability, including attention and respond control, before and after each treatment course of EEG neurofeedback in this study. Six comprehensive quotients, including the full scale response control quotient (FRCQ), auditory response control quotient (ARCQ), visual respond control quotient (VRCQ), full scale attention quotient (FAQ), auditory attention quotient (AAQ), and visual attention quotient (VAQ), as well as six respond control scale quotients, including prudence auditory (PRUA), prudence vision (PRUV), consistency auditory (CONA), consistence vision (CONV), stamina auditory (STAA) and stamina vision (STAV), and six attention scale quotients, including vigilance auditory (VIGA), vigilance vision (VIGV), focus auditory (FOCA), focus vision (FOCV), speed auditory (SPDA), and speed vision (SPDV) were assessed. Among these three types of quotients, attention and respond control ability quotients were basic, and comprehensive quotients were calculated from basic quotients. The basic and comprehensive quotients had different emphases, that is, the former quotients were single correlation quotients that reflected a single skill, whereas the latter quotients comprehensively evaluated the control ability of subjects.

### 2.4 Quality control

Our study was carried out by a well‐trained research staff along with doctors, and they were trained before the study. The training content included purpose and procedures of the study and how to implement the tests. The instruments used in the trial were standardized by the local technical supervision bureau. Patients were diagnosed and classified by two physicians with the rank of associate chief or above.

Before treatment, subjects were given training guidance to master the principles of brain waves and the target task of neurofeedback. A health education program was also administered to parents of subjects. The purpose and significance of treatment were clarified three times so that they could supervise and urge subjects to cooperate during the treatment.

### 2.5 Statistical analysis

All data were collected and analyzed by use of SPSS software (version 22.0 SPSS Inc., Chicago, IL, USA). Numerical variables were expressed as mean ± standard deviation. Age, IQ, and the sex ratio of children between ADHD‐PI and ADHD‐CT groups were compared by the independent sample t test and Chi‐square test, respectively. The changes or differences in IVA/CPT quotients and brain waves among each treatment course or between two groups were analyzed by repeated measures analysis (general linear model). The Greenhouse–Geisser corrected F test (calibration degree of freedom ranged from 1 to 3) was used when the data did not conform to Mauchly's test of sphericity. The Bonferroni test was used for comparisons of the confidence interval of main effect, and the F test was used for interactions between two groups and indexes among each course of treatment. All tests were two‐tailed, and *p *< .05 was considered statistically significant.

## 3 RESULTS

### 3.1 Age, gender, and IQ of subjects

Before treatment, age, gender and IQ of children between two groups were balanced and comparable. All subjects were aged from 6.02 to 11.78 years, with a mean of 8.29 ± 1.46 years, and there was no significant difference between ADHD‐PI (8.06 ± 1.28) and ADHD‐CT (8.36 ± 1.51, *t* = .664, *p *= .511). There were 32 boys and six girls, the sex ratio was 16:3, and there was no significant difference in the sex ratio between ADHD‐PI (15:4) and ADHD‐CT (17:2, *χ* ^2^ = .792, *p *= .374) groups. The mean IQ of all subjects was 101.81 ± 12.79, and there was no significant difference in the IQ of subjects between ADHD‐PI (101.74 ± 14.76) and ADHD‐CT (100.84 ± 11.25, *t* = .210, *p *= .835) groups.

### 3.2 The relative power and ratio of brain waves in the two groups changed significantly after three treatment courses

**FIGURE 1:**
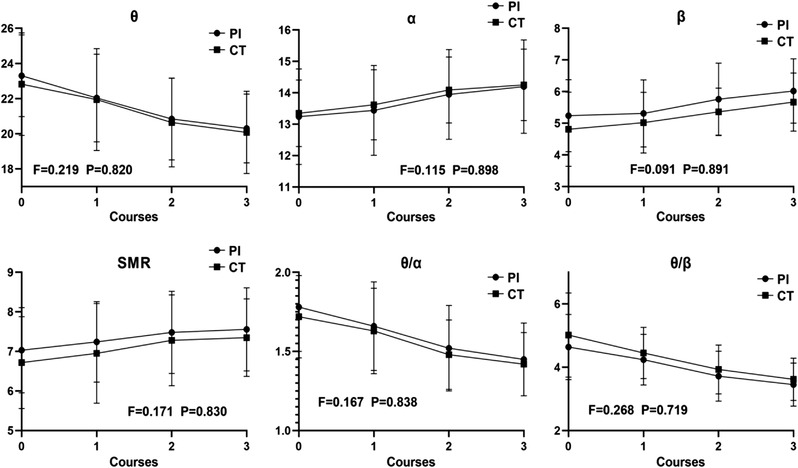
Relative power and ratio of brain waves in both types before and after each course

The interactions between different ADHD types and treatment courses had no significant effects on *θ*, *α*, *β*, SMR, *θ*/*α*, and *θ*/*β* (all *p *> .05, Figure 1). Therefore, it was necessary to interpret main effects of treatment course.

There were significant differences in main effects of treatment course in the relative power and ratio of brain waves in both types (all *p *< .05, Table 1).

**TABLE 1:** Comparison of relative powers and ratios of brain waves before and after every treatment course in each type $\left(\bar{x}\pm s\right)$

Index	Type	Before treatment	One course	Two courses	Three courses	*F*	*p*
*θ*	ADHD‐PI	23.30 ± 2.33	22.04 ± 2.49^a^	20.84 ± 2.33^b^	20.30 ± 1.96^b,c^	22.378	.000
	ADHD‐CT	22.82 ± 2.92	21.95 ± 2.90^a^	20.64 ± 2.52^b,d^	20.08 ± 2.34^b,^ ^d^	33.679	.000
*α*	ADHD‐PI	13.24 ± 1.52	13.44 ± 1.43	13.95 ± 1.43^b,^ ^d^	14.20 ± 1.49^b,^ ^d^	19.289	.000
	ADHD‐CT	13.35 ± 1.06	13.62 ± 1.12	14.09 ± 1.05^b^	14.25 ± 1.14^b,c^	12.435	.000
*β*	ADHD‐PI	5.24 ± 1.14	5.31 ± 1.06	5.76 ± 1.14	6.02 ± 1.02^a,c^	5.043	.014
	ADHD‐CT	4.81 ± 1.17	5.02 ± 0.96	5.36 ± 0.75	5.67 ± 0.92^b,^ ^d^	10.250	.000
SMR	ADHD‐PI	7.03 ± 1.08	7.24 ± 1.02	7.48 ± 1.04	7.56 ± 1.05^a^	6.451	.011
	ADHD‐CT	6.72 ± 1.16	6.95 ± 1.26	7.28 ± 1.15	7.35 ± 0.98	4.403	.023
*θ*/*α*	ADHD‐PI	1.78 ± 0.28	1.66 ± 0.28	1.52 ± 0.27^b,^ ^d^	1.45 ± 0.23^b,^ ^d^	20.822	.000
	ADHD‐CT	1.72 ± 0.26	1.63 ± 0.27	1.48 ± 0.22^b,^ ^d^	1.42 ± 0.20^b,^ ^d^	27.698	.000
*θ*/*β*	ADHD‐PI	4.64 ± 1.03	4.24 ± 0.87	3.72 ± 0.79^a,c^	3.45 ± 0.68^b,^ ^d^	16.383	.000
	ADHD‐CT	5.02 ± 1.33	4.45 ± 0.81	3.93 ± 0.77^b,^ ^d^	3.62 ± 0.67^b,^ ^d^	21.012	.000

^a^ *p *< .05 compared to before treatment.

^b^ *p *< .01 compared to before treatment.

^c^ *p *< .05 compared to after one treatment course.

^d^ *p *< .01 compared to after one treatment course.

After one treatment course of EEG neurofeedback, *θ* in both types decreased significantly (all *p *< .05).

Compared to before treatment and after one treatment course, *θ*/*α* and *θ*/*β* in both types, *θ* in ADHD‐CT decreased significantly, whereas *α* in ADHD‐PI increased after two treatment courses of EEG neurofeedback (all *p *< .05). Similarly, after two treatment courses of EEG neurofeedback, *θ* in ADHD‐PI decreased significantly and *α* in ADHD‐CT increased significantly compared to before treatment (all *p *< .01). The data are presented in Table 1.

After three treatment courses of EEG neurofeedback, compared to before treatment and after one treatment course, *θ*/*α*, *θ*/*β*, and *θ* in both types decreased significantly, whereas *α* and *β* in both types increased (all *p *< .05). In addition, SMR in ADHD‐PI was significantly higher than that before treatment (all *p *< .05, Table 1). However, there were no significant differences in the relative power and ratio of brain waves in both types between two and three treatment courses.

### 3.3 Most IVA/CPT quotients in the two types increased significantly after three treatment courses

**FIGURE 2:**
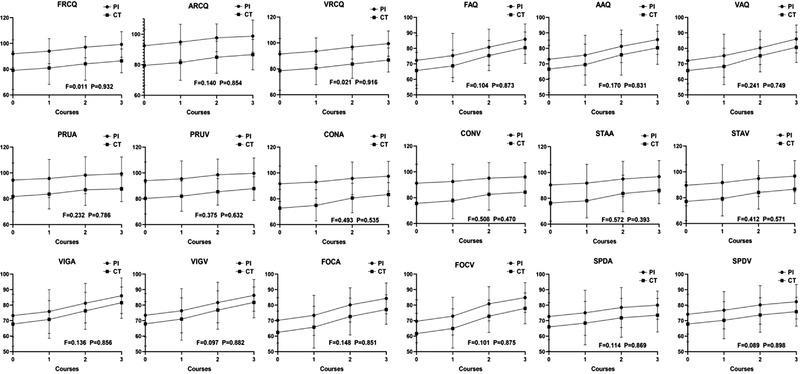
Comparison of IVA‐CPT quotients between two types at each time point

The interactions between different ADHD types and treatment courses had no significant effect on all 18 IVA/CPT quotients (all *p *> .05, Figure 2). Therefore, it was necessary to interpret main effects of treatment course.

There were significant differences in the main effects of the treatment course in IVA/CPT quotients in both types (all *p *< .05, Table 2).

**TABLE 2:** Comparison of IVA/CPT quotients before and after every treatment course in each type $\left(\bar{x}\pm s\right)$

Quotient	Type	Before treatment	One course	Two courses	Three courses	*F*	*p*
FRCQ	ADHD‐PI	92.05 ± 10.81	94.05 ± 9.78	97.11 ± 8.31	99.26 ± 9.98^a^	6.698	.006
	ADHD‐CT	79.11 ± 14.76	80.95 ± 12.18	84.16 ± 12.71	86.47 ± 9.25^a^	4.267	.023
ARCQ	ADHD‐PI	92.47 ± 10.61	94.84 ± 11.66	97.63 ± 9.10^a^	98.79 ± 10.53^a^	7.105	.001
	ADHD‐CT	79.58 ± 13.15	81.47 ± 11.73	84.95 ± 10.57^b^, ^c^	86.63 ± 9.98^b^, ^d^	15.511	.000
VRCQ	ADHD‐PI	91.47 ± 11.92	93.63 ± 10.19	96.84 ± 9.25^a^	99.47 ± 9.77^a^	5.023	.021
	ADHD‐CT	78.54 ± 15.04	80.58 ± 12.76	83.74 ± 11.09^b^, ^c^	86.84 ± 9.25^b^, ^d^	16.085	.000
FAQ	ADHD‐PI	72.26 ± 16.01	75.37 ± 14.28	80.79 ± 11.69^a^	85.89 ± 9.89^b,^ ^d^, ^e^	14.206	.000
	ADHD‐CT	65.74 ± 11.69	68.74 ± 10.22^a^	75.37 ± 9.76^b^, ^d^	80.42 ± 10.16^b^, ^d^, ^e^	27.430	.000
AAQ	ADHD‐PI	72.96 ± 12.41	75.63 ± 12.85	81.32 ± 10.51^b^	85.68 ± 9.59^b^, ^d^	18.980	.000
	ADHD‐CT	66.63 ± 15.27	69.47 ± 13.26^a^	75.84 ± 13.11^b^, ^d^	80.37 ± 10.67^b^, ^d^, ^e^	18.864	.000
VAQ	ADHD‐PI	72.11 ± 14.96	75.26 ± 13.92^a^	80.32 ± 10.87^b,c^	86.05 ± 9.03^b^, ^d^, ^e^	19.832	.000
	ADHD‐CT	65.68 ± 12.65	68.37 ± 11.77^a^	75.21 ± 10.38^b,^ ^d^	80.68 ± 9.79^b^, ^d^ ^,d,e^	41.173	.000
PRUA	ADHD‐PI	94.53 ± 13.23	95.74 ± 14.71	98.27 ± 14.30	99.38 ± 12.95	3.004	.103
	ADHD‐CT	81.74 ± 11.79	83.62 ± 11.52	86.93 ± 12.11^a^	87.76 ± 10.03^a^	5.428	.018
PRUV	ADHD‐PI	94.03 ± 14.41	95.42 ± 13.85	98.56 ± 12.24	99.73 ± 11.89	3.147	.085
	ADHD‐CT	80.26 ± 12.13	82.05 ± 11.70	85.42 ± 10.28^a^	87.89 ± 9.19^b,c^	8.428	.003
CONA	ADHD‐PI	91.62 ± 13.76	92.98 ± 12.54	95.78 ± 12.63	97.46 ± 11.58^a^	4.133	.039
	ADHD‐CT	72.71 ± 14.61	74.86 ± 12.06	80.57 ± 11.40^b^, ^d^	83.34 ± 9.18^b^, ^d^	13.752	.000
CONV	ADHD‐PI	91.20 ± 14.68	92.56 ± 13.38	95.12 ± 12.10	96.19 ± 11.07	3.106	.093
	ADHD‐CT	75.57 ± 15.31	77.62 ± 13.97	82.56 ± 12.23^b^, ^d^	84.29 ± 10.77^b^, ^d^	12.166	.000
STAA	ADHD‐PI	90.33 ± 15.61	91.79 ± 14.57	94.95 ± 13.72^a^	96.75 ± 12.49^b^	4.855	.027
	ADHD‐CT	76.36 ± 14.09	78.09 ± 13.34	83.71 ± 14.17^b^, ^d^	86.19 ± 10.37^b^, ^d^	14.732	.000
STAV	ADHD‐PI	89.60 ± 15.89	91.64 ± 13.94	94.87 ± 13.87^a^	96.69 ± 12.09^b^, ^c^	5.185	.020
	ADHD‐CT	77.14 ± 14.98	79.33 ± 13.45	84.07 ± 12.89^b^, ^d^	86.60 ± 11.18^b^, ^d^	11.775	.000
VIGA	ADHD‐PI	73.27 ± 13.76	75.91 ± 14.09	81.29 ± 12.74^b^, ^c^	86.07 ± 11.52^b^, ^d^, ^e^	13.201	.000
	ADHD‐CT	67.82 ± 14.39	70.76 ± 12.15	76.31 ± 11.98^b^, ^d^	81.57 ± 10.15^b^, ^d^, ^e^	18.910	.000
VIGV	ADHD‐PI	73.49 ± 13.95	76.35 ± 14.28^a^	81.67 ± 13.07^b^, ^d^	86.33 ± 10.26^b^, ^d^, ^e^	15.440	.000
	ADHD‐CT	67.93 ± 14.32	71.02 ± 13.62^a^	76.83 ± 12.39^b,d^	81.76 ± 9.95^b^, ^d^, ^e^	17.836	.000
FOCA	ADHD‐PI	70.18 ± 14.59	73.39 ± 12.92^a^	80.13 ± 11.04^b^, ^d^	84.37 ± 9.98^b^ ^,d,^ ^e^	17.863	.000
	ADHD‐CT	62.43 ± 13.72	65.78 ± 13.51^a^	72.64 ± 11.89^b^, ^d^	77.13 ± 9.54^b^, ^d^, ^e^	26.942	.000
FOCV	ADHD‐PI	69.62 ± 13.91	72.89 ± 12.23^a^	80.87 ± 11.04^b^, ^d^	84.90 ± 9.69^b^, ^d^, ^e^	19.194	.000
	ADHD‐CT	61.64 ± 13.09	64.93 ± 12.67^a^	72.84 ± 10.46^b^, ^d^	77.93 ± 9.96^b^, ^d^, ^e^	36.905	.000
SPDA	ADHD‐PI	72.73 ± 14.95	75.09 ± 14.58	78.53 ± 12.93^b^, ^c^	80.07 ± 9.10^b^, ^c^	6.951	.006
	ADHD‐CT	66.03 ± 15.69	68.43 ± 14.02	71.86 ± 12.54^b^, ^c^	73.55 ± 10.83^b^, ^c^	5.722	.015
SPDV	ADHD‐PI	74.13 ± 12.83	76.75 ± 12.08	80.19 ± 12.41^b^ ^,c^	82.27 ± 11.07^b^, ^d^	7.836	.005
	ADHD‐CT	67.87 ± 11.52	70.28 ± 11.96	73.67 ± 11.14^b^, ^c^	75.84 ± 9.36^b^, ^d^	8.217	.004

^a^ *p *< .05 compared to before treatment.

^b^ *p *< .01 compared to before treatment.

^c^ *p *< .05 compared to after one treatment course.

^d^ *p *< .01 compared to after one treatment course.

^e^ *p *< .05 compared to after two treatment courses.

After one treatment course of EEG neurofeedback, VAQ, VIGA, FOCA, and FOCV in both types, as well as FAQ and AAQ in ADHD‐CT increased significantly (all *p *< .05). The data are presented in Table 2.

VAQ, VIGA, VIGV, FOCA, FOCV, SPDA, and SPDV in both types, as well as ARCQ, VRCQ, FAQ, AAQ, CONA, CONV, STAA, and STAV in ADHD‐CT after two treatment courses of EEG neurofeedback were significantly higher than those before treatment and after one treatment course (all *p *< .05). PRUA and PRUV in ADHD‐CT, as well as ARCQ, VRCQ, FAQ, AAQ, STAA, and STAV in ADHD‐PI after two treatment courses of EEG neurofeedback were significantly higher than those before treatment (all *p *< .05). The data are presented in Table 2.

As shown in Table 2, FAQ, VAQ, VIGA, VIGV, FOCA, and FOCV in both types, as well as AAQ in ADHD‐CT after three treatment courses of EEG neurofeedback were significantly higher than those after two treatment courses (all *p *< .05). FAQ, AAQ, VAQ, STAV, VIGA, VIGV, FOCA, FOCV, SPDA, and SPDV in both types, as well as ARCQ, VRCQ, PRUV, CONA, CONV, and STAA in ADHD‐CT after three treatment courses of EEG neurofeedback were significantly higher than those before treatment and after one treatment course (all *p *< .05). FRCQ and PRUA in ADHD‐CT, as well as FRCQ, ARCQ, VRCQ, CONA, and STAA in ADHD‐PI after three treatment courses of EEG neurofeedback were significantly higher than those before treatment (all *p *< .05).

## 4 DISCUSSION

In the connective process of brain function, neural resource allocation is different when attention is used for task control (Hale et al., 2014). Task execution would be interfered when the connectivity of brain function decreases, and then cognitive function is impaired (Silberstein et al., 2016). The change in EEG power can affect the regulation of neural network. Compared with the absolute power value of EEG, the relative power value of EEG tends to be more stable and easier to quantify. Johnstone et al. (2017) compared the relative power value of EEG before and after neurofeedback in ADHD and found that the δ wave decreased, while the *α* wave increased. In our study, during whole three‐course structural paradigm treatment of EEG neurofeedback, *θ* in both types decreased gradually with the extension of treatment time. In addition, *θ*/*β* and *θ*/*α* in both types showed a downward trend after two and three treatment courses. By contrast, *α* and *β* in both types increased significantly after two and three treatment courses. These results indicated that both structural patterns of EEG neurofeedback for ADHD‐PI and ADHD‐CT patients were effective. In addition, changes in EEG were observed after two treatment courses, and the curative effect showed an obviously increasing trend with the prolongation of treatment course, suggesting that at least two consecutive treatment courses are required so as to achieve relatively satisfactory therapeutic effects.

CPT is applied to evaluate the continuous attention and reaction control of cognitive function, and it is basically free from subjective factors. Researchers have already demonstrated the feasibility of CPT as an assessment and diagnostic tool of ADHD (Gilbert et al., 2016). IVA/CPT quotients quantify and standardize the core symptoms of ADHD. IVA/CPT quotients measure the sustained attention of visual and auditory response through random appearance of audio‐visual mixed signals to identify the random response, impulse conflict, and fatigue status (Simões et al., 2017). These factors can be judged by quantitative scores (quotients). The integration of IVA/CPT quotients improved ADHD diagnosis and better reflected complexity and heterogeneity of ADHD (Berger et al., 2017). In this study, after two treatment courses, besides PRUA, PRUV, CONA, CONV in ADHD‐PI and FRCQ in both types, other IVA/CPT quotients in both types increased significantly. In response to this, most symptoms of ADHD patients were relieved. After three treatment courses, in addition to CONA in ADHD‐PI, FRCQ in both types also increased significantly. FAQ, VAQ, VIGA, VIGV, FOCA, FOCV in both types, and AAQ in ADHD‐CT were significantly higher than those after two treatment courses. And along with this, the symptoms of ADHD patients improved further during the third treatment course. This change trend of IVA/CPT quotients with the increase of treatment course was consistent with the change rule of EEG neurofeedback, that is, the potential function of each brain wave needs to be stimulated and used rationally in mental applications and subsequently stabilized through long‐term and multi‐course training. Therefore, compared with the results of previous studies, which mostly applied two treatment courses, our study further indicated the necessity for consistent three‐course EEG neurofeedback in the treatment of ADHD.

Six attention quotients, namely vigilance, focus, and speed quotients of visual and auditory signals, were used to measure the errors of omission, sensitivity to change, and reaction speed during the test, respectively. Taken collectively, they reflect the attention problems associated with slow mental activity or the attention deficit of subjects. In this study, from the second treatment course of EEG neurofeedback, vigilance, focus, and speed quotients in both types increased unanimously. Meanwhile, the three comprehensive quotients reflecting attention ability (FAQ, AAQ, and VAQ) in both types increased gradually. Namely, subjects’ attention improved significantly after two treatment courses, and further improved and stabilized after three treatment courses. The results also indicated that different structural patterns of the EEG neurofeedback targeted for different ADHD types could improve the attention retention ability and concentration of patients with different ADHD types, and the therapeutic effects are basically same.

Six control quotients, namely prudence, consistency and stamina quotients of visual and auditory signals, were used to measure the ability to stop, think, recognize, respond correctly to disturbances, maintain the response consistency over time, and remain stable throughout the test, respectively. They jointly reflect the overall coordination and volitional control abilities of the subjects. In ADHD‐CT, prudence, consistency, and the stamina quotients of children increased significantly after two treatment courses, revealing that the ability to deal with disturbance and attention control of these children improved dramatically. And further improvement and stabilization of these two abilities were achieved after three treatment courses. In ADHD‐PI, only the stamina quotients of children increased after two treatment courses. This was reasonable because the ability to deal with disturbance of external stimuli or information and attention control of ADHD‐PI patients was still within normal range. And after each treatment course, the corresponding quotients reflecting these two abilities did not change significantly. Above results together suggested that different structural patterns of EEG neurofeedback targeted for characteristics of brain function of different ADHD types was necessary and feasible. In the other hand, three comprehensive quotients reflecting the respond control ability (FRCQ, ARCQ, and VRCQ) in both types improved, except these changes were more obvious in ADHD‐CT. Taken collectively, these results indicate that different structural patterns of EEG neurofeedback targeted for different ADHD types adopted in this study can effectively improve the cognitive and executive functions of different types of ADHD patients. And the more treatment courses, the better treatment effect.

This study had some limitations. The sample size of this study was comparatively small and only met the basic statistical requirements. The statistical efficiency would have improved if blind grouping was used and/or the sample size was increased appropriately. Furthermore, all subjects were from Jiaxing City. As a medium‐sized city, although Jiaxing City has a good representative, there might have regional limitations. Therefore, further studies are needed to verify the results of this study.

## 5 CONCLUSION

In summary, the results of this study indicated that different structural patterns of EEG neurofeedback targeted for ADHD‐CT and ADHD‐PI were both effective and feasible, especially in cognitive and executive functions, including attention and respond control abilities. Two treatment courses of EEG neurofeedback had a good therapeutic effect in ADHD patients, but three treatment courses were most effective. In the future, this targeted and different structural pattern multi‐course treatment of EEG neurofeedback deserves to be applied in the treatment of ADHD to achieve a better therapeutic effect.

## CONFLICT OF INTEREST

No financial or non‐financial benefits have been received or will be received from any party related directly or indirectly to the subject of this article.

## AUTHOR CONTRIBUTORS

*Concept and design*: Tong‐kun Shi. *Methodology*: Xiao‐mei Cui and He‐dan Zhao. *Data curation*: Ling‐fei Yang and Zheng Wang. *Writing—original draft*: Feng‐hua Wang and Li‐yan Sun. *Writing‐review and editing*: Feng‐hua Wang and Li‐yan Sun. *Project Administration*: Tong‐kun Shi. *Funding acquisition*: Tong‐kun Shi. All authors have read and agreed to the published version of the manuscript.

### PEER REVIEW

The peer review history for this article is available at https://publons.com/publon/10.1002/brb3.2572

## Data Availability

The data presented in this study are available upon request from the corresponding author.
